# Seismic slope stability analysis using modified pseudo dynamic method with uniform random field of initial phases

**DOI:** 10.1371/journal.pone.0330435

**Published:** 2025-08-22

**Authors:** Liang Xu, Hongliang Jing, Jiahui Wen, Liang Li, Ziyang Song

**Affiliations:** School of Civil Engineering, Qingdao University of Technology, Qingdao, China; China University of Mining and Technology, CHINA

## Abstract

This paper develops an enhanced pseudo dynamic method for conducting seismic stability analysis of slopes. The enhancement is justified by the development of uniform sampling approach and the incorporation of both different and correlated initial phases through a uniform random field. The advanced methodology, named by modified pseudo dynamic Bishop method (MPDBM) is verified against the existing research results and extended to homogeneous soil slope with different scales emphasizing the effect of scale of fluctuation on the minimum factor of safety (FS_min_). The numerical results demonstrate that the identical assumption on initial phases in the traditional pseudo dynamic method underestimates the FS_min_ as compared to the current method considering the spatial variability of initial phases for the cases where the slope soil is subjected to its natural frequency. This underestimation grows significant as the slope scale enlarges.

## 1. Introduction

Slope failures caused by earthquakes have led to huge economic losses and casualties, and hence seismic slope stability analysis has triggered the worldwide attention [1–7]. Among the methods for seismic slope stability evaluation, the Factor of Safety (FS)-based one still remains fair popularity owing to its simplicity and easiness in decision making on slope stability. The FS-based methodology can be further classified into three categories: (1) pseudo static method(PSM), in which seismic forces are dealt with by constant forces acting on the centriod of sliding mass [8–10]; (2) pseuso dynamic method (PDM) where part of the dynamic characteristics of seismic forces are taken into account [11–14]; and (3) dynamic time history method, in which the full dynamic characteristics of seismic forces are properly considered. To put it in detail, the PSM, by adding an equivalent static force loaded at the center of sliding mass to the conventional limit equilibrium method, results in a pseudo static Factor of Safety(FS), based on which the seismic stability of slopes can be evaluated in a simplified manner. The PSM finds overwhelming popularity within the geotechnical practitioners, although it only focuses on the amplitude of the seismic force. The dynamic time history method, integrates the complicated soil dynamic constitutive model and the seismic acceleration records to obtain the distributions of stress, strain, and water pressure within the sliding mass, based on which the comprehensive assessment on seismic stability of slopes can be achieved in a systematic but inefficient manner as compared to PSM. As a proper balance between PSM and dynamic time history method, the PDM, mimics the seismic force using a sinusoidal wave stemming from the slope base with a specific initial phase [15,16] to properly address the dynamic characteristic of earthquake.

The PDM was originated by Steedman and Zeng and was validated against the seismic active thrust on a rigid retaining wall [15]. Unlike the constant or linearly varied seismic forces acting on the vertical components of sliding mass in pseudo static method, the concept of initial phase was introduced in PDM to address the variation of seismic forces acting on the vertical components of sliding mass. The seismic acceleration is regarded as a sinusoidal wave projecting from the slope base with a specific initial phase and the seismic acceleration acting on each of vertical components of the sliding mass can be directly determined with the propagation velocity of the sinusoidal wave in the original PDM [17]. The original concept of PDM was integrated with limit equilibrium method and limit analysis to evaluate the seismic slope stability [18–23]. For example, Pan et al. [18] calculated the reliability of slopes using limit analysis coupled with PDM highlighting the influence of slope geometry and seismic parameters on the probability of failure. Hazari et al. [24] combined the Swedish circle method with PDM to examine the slope stability and compared the results from the proposed method with those by the finite element method. Zhou and Qin [11] used the upper bound limit analysis method coupled with PDM to solve the FS of slopes emphasizing on the linear amplification on the amplitude of seismic acceleration.Their outputs have demonstrated that the PDM leads to larger FS than PSM does indicating that the PSM tends to underestimate the FS of the slope and thus to make conservative decision on slope stability. It was also noted in [11] that the increase in FS form the PDM fades out as the shear wave velocity increases, which was also confirmed by [23]. Despite the popularity of original PDM, it suffers from the incompatible stress boundary condition at the slope crest. To properly address this issue, Bellezza [16] developed a modified pseudo dynamic method(MPDM) by regarding the slope soil as a more realistic visco-elastic material(e.g., Kelvin-Voigt material represented by a purely elastic spring and a purely viscous dashpot connected in parallel). Since the origination of MPDM, it has been applied into seismic-related geotechnical problems. For example, Chanda et al [25] proposed a method for calculating slope stability based on MPDM using the horizontal slice method. Zhong and Yang [19] introduced a method for analyzing the stability of rock slopes using the MPDM within the framework of limit analysis.Zhou and Qin [26] analyzed the impact of soft bands on the seismic stability of slopes using finite element limit analysis combined with MPDM. Li et al. [27] used the upper bound theorem of limit analysis to analyze the stability of slopes reinforced with piles under the MPDM, finding that the FS of slope decreases significantly when the normalized frequency of the shear wave coincides with the natural frequency of the soil, which agrees well with that presented in [16].

The literature review on the PDM and the MPDM has found that the FS from the PDM and MPDM is dependent on the initial phase and the most dangerous initial phase must be determined to obtain the minimum FS (denoted herein by FS_min_), based on which the slope stability is evaluated by PDM and MPDM in the previous studies. However, it must be noted that the initial phase at different locations of slope base is assumed to be identical in the existing studies. This assumption may be applicable to small scale slopes. However, for large scale slopes, the initial phase at the slope base may be intuitively different but correlated within one seismic motion regarding the influencing factors as epicentral distance, epicenter depth, and the propagation velocity of the seismic wave. In addition, the seismic slope stability was investigated considering the different arrival times of seismic waves at various locations [28–30], which justified the necessity of incorporating the different but correlated initial phase in seismic slope stability using PDM or MPDM. Therefore, how to evaluate the seismic slope stability considering both the difference and correlation in initial phase using MPDM remains an open question.

This paper begins with the combination of MPDM with simplified Bishop Method, followed by the simulation of initial phases using random field theory. Then, the proposed methodology, named modified pseudo dynamic Bishop method(MPDBM) is described in detail and is validated against the previous research outputs. Then, The proposed methodology is elucidated through homogeneous soil slopes with different scales to investigate the effect of damping ratio, scale of fluctuation, and slope scale on the seismic slope stability. Finally, discussions and conclusions are drawn based on the findings.

## 2. The pseudo dynamic Bishop method for seismic slope stability

### 2.1 Calculation of horizontal seismic force by MPDM

**Fig 1:**
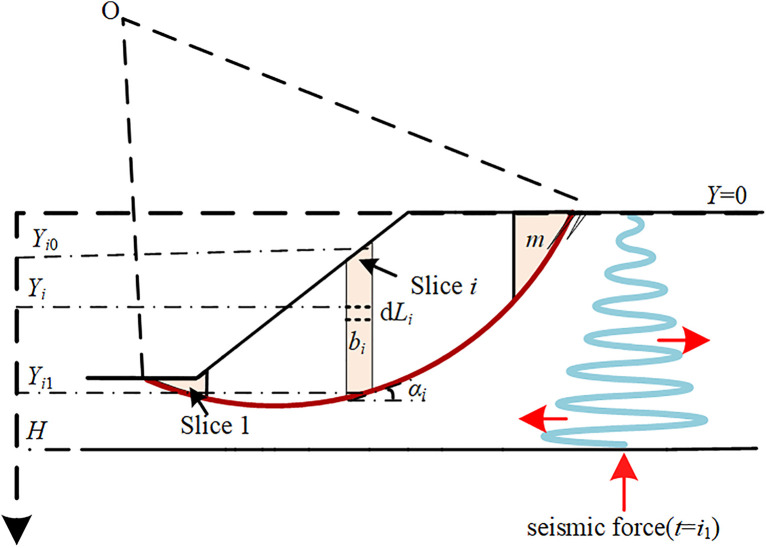
The diagram of horizontal seismic force in MPDM.


The MPDM developed in [16] is adopted herein to calculate the horizontal seismic force acting on the sliding mass. Fig 1 demonstrates the distribution of horizontal seismic force, which is proportional to the distribution of propagated acceleration with initial phase *t* = *i*_1_ at the slope base. The selected circular sliding surface has a center point of O and radius of *R*. The sliding mass is composed of the soils between the slope surface in black line and circular sliding surface in red line as shown in Fig 1. The sliding mass is divided into a finite number(e.g., *m* in Fig 1) of vertical slices for the easy implementation of limit equilibrium method. Define an axis *Y* with origin at the slope crest and its positive direction is downward. The *Y* coordinate of the slope base is *H.* Consider slice *i* for example, *b*_*i*_ is the width of slice *i*; *γ*_*i*_ is the average unit weight of the slice *i*; *α*_*i*_ is the inclination angle of slice *i* with respect to horizontal direction. An arbitrary horizontal sub-element of slice *i* with *Y* coordinate of *Y*_i_ has a height of d*Y*_*i*_ and a width of *b*_*i*_. *Y*_*i*0_ and *Y*_*i*1_ respectively denote the *Y* coordinates of top and bottom centers of slice *i*; Following the assumptions adopted by MPDM, the arrived seismic motion is subsequently propagated through the visco-elastic slope soils in a wave like that shown in pink. Note that the angular frequency of seismic motion is *ω* and the propagation velocity of the shear wave in the slope soil is *V*_s_. The damping ratio of soil is *D.* The horizontal seismic force acting on the aforementioned sub-element of slice *i*, denoted by *F*_h*i*_(*t*), is:

$$F_{hi}(t)=\gamma_{i}b_{i}\frac{k_{h}\times g}{Cs^{2}+Ss^{2}}[(CsC_{sz}+SsS_{sz})\cos(\omega t)+(SsC_{sz}-CsS_{sz})\sin(\omega t)]dY_{i}$$ (1)

where *k*_h_ is the horizontal seismic coefficient at the slope base; Four variables, denoted by C_sz_,S_sz_,Cs,Ss are defined to facilitate the expression of Eq. (1). They are dependent on the *ω*, *D*, *V*s, and *Y*i.


$$C_{sz}=\cos(k_{s1}Y_{i})\cosh(k_{s2}Y_{i})$$ (2)

$$S_{sz}=-\sin(k_{s1}Y_{i})\sinh(k_{s2}Y_{i})$$ (3)

$$Cs=\cos(k_{s1}H)\cosh(k_{s2}H)$$ (4)

$$Ss=-\sin(k_{s1}H)\sinh(k_{s2}H)$$ (5)

where *k*_s1_ and *k*_s2_ are defined as:


$${k_{s}}_{1}=\frac{\omega }{V_{s}}\sqrt{\frac{\sqrt{1+4D^{2}}+1}{2(1+4D^{2})}}$$ (6)

$$k_{s2}=-\frac{\omega }{V_{s}}\sqrt{\frac{\sqrt{1+4D^{2}}-1}{2(1+4D^{2})}}$$ (7)

The resultant sliding moment acting on the slice *i* from the horizontal seismic force can be determined by integration:


$$M_{i}(t)=\int_{Y_{i0}}^{Y_{i1}}[R\times \cos\alpha_{i}+Y_{i}-Y_{i1})]\times F_{hi}(t)$$ (8)

where $M_{i}(t)$ is the sliding moment acting on the slice *i* from the horizontal seismic force. The resultant sliding moment acting on the whole sliding mass can be summed as:


$$M(t)=\sum_{i=1}^{m}M_{i}(t)$$ (9)

### 2.2 Pseudo Dynamic Bishop Method(PDBM) for slope stability

**Fig 2:**
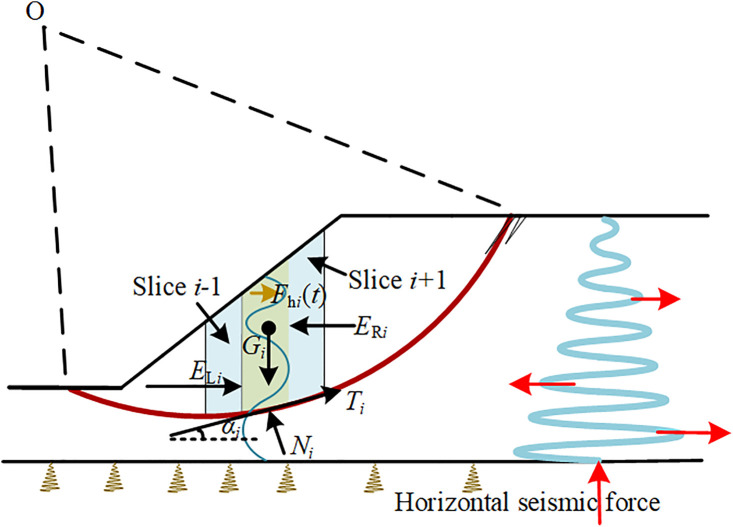
Forces acting on slice *i.*

**T**he traditional simplified Bishop method [31] can be extended to a pseudo dynamic Bishop method for slope stability, where the contribution of horizontal seismic force is quantified using the MPDM as described in Eq.(9). Refer to Fig 2, where the sliding soil is divided into *m* slices. *G*_*i*_ is the weight of slice *i*, *E*_R*i*_ is the horizontal inter-slice force between slice *i* and slice *i* + 1, *E*_L*i*_ is the horizontal interslice force between slice *i* and slice *i*-1,*N*_*i*_ and *T*_*i*_ are respectively the normal and shear force at the base of slice *i*, *F*_h*i*_(*t*) is the distributed horizontal seismic force. Thanks to the horizontal direction for the *F*_h*i*_(*t*), the force equilibrium equation in vertical direction in traditional simplified Bishop method can still be available.

Hence, the traditional simplified Bishop method is extended by directly incorporating the contributions of sliding moment resulting from *F*_h*i*_(*t*). The PDBM for slope stability is:


$$FS(t)=\frac{\sum_{i=1}^{m}\frac{\left(G_{i}\times \tan\varphi_{i}+c_{i}b_{i}\right)}{\cos\alpha_{i}(1+\tan\alpha_{i}\tan\varphi_{i}/FS(t))}\times R}{\sum_{i=1}^{m}\left(G_{i}\times \sin\alpha_{i}\times R+M_{i}(t)\right)}$$ (10)

where FS(*t*) is the Factor of Safety at initial phase *t*; *φ*_*i*_ and *c*_*i*_ is the internal friction angle and cohesion at the base of slice *i*.

**Fig 3:**
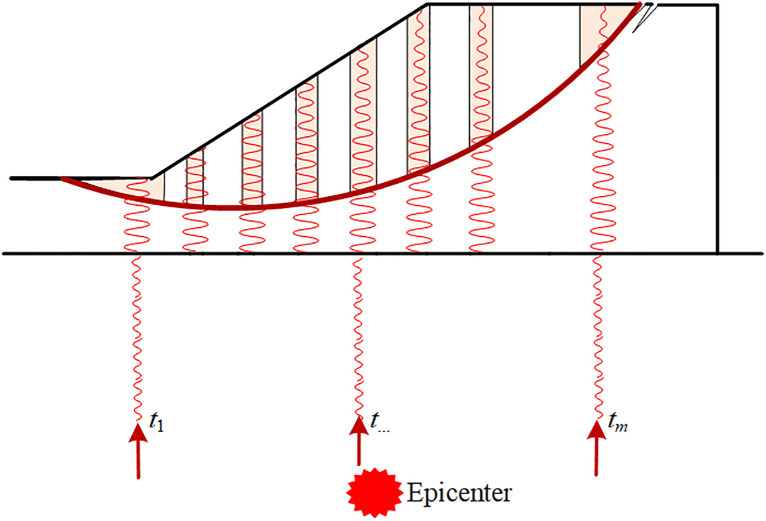
Schematic diagram of identical assumption on initial phases.

**Fig 4:**
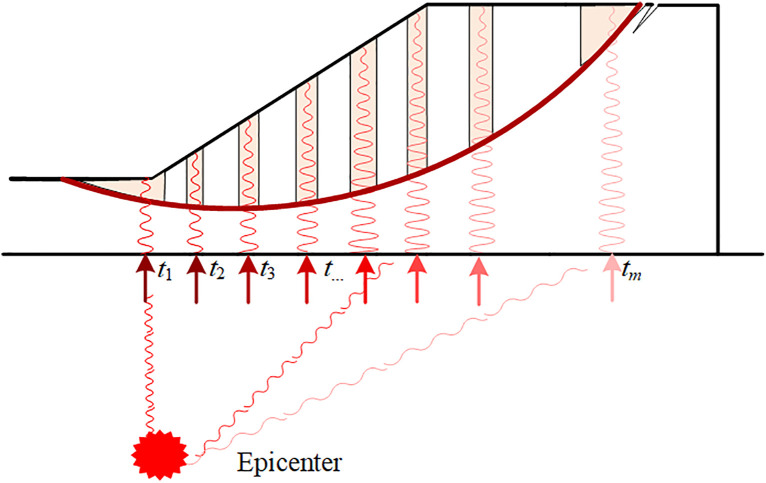
Schematic diagram of different initial phases.

Obviously, the calculation of FS using the PDBM depends on initial phase *t*, as indicated by Eq.(10). Previous studies [13,23] have to alter the initial phase *t* and to find the FS_min_ of PDM or MPDM for seismic slope stability. However, the initial phase *t*s at different locations(e.g., *t*_1_, *t*_2_, and *t*_*m*_ as shown in Fig 3) on the slope base are assumed to be identical(e.g., *t*_1_ = *t*_2_ = …, *t*_*m*_ = *t*). This identical assumption is applicable to the small scale slope where the slope width is negligible as compared to the epicentral distance. As will be discussed in case studies, the initial phase at different locations on the slope base may be varied for medium and large scale slopes. As compared to Fig 3, Fig 4 shows the seismic force distribution of a slope in the case of different initial phases. The arrows below the slope base in different colors indicate the different initial phases of the seismic wave that reaches the base. The comparison between the seismic force distribution of the sliding mass illustrates the difference between identical assumption (used in PDM and MPDM) and different initial phase assumption(will be considered in this study) justifies the necessity of incorporating variations in initial phase in seismic slope stability using PDM and MPDM.

## 3. The simulation of both different and correlated initial phases

### 3.1 Influencing factors of both different and correlated initial phases

In this section, the influences of epicentral horizontal distance(denoted by *E*_h_), the epicentral depth(denoted by *E*_d_), and the propagation velocity of seismic wave within the slope base(denoted by *V*_p_) are demonstrated and discussed in detail for the further understanding of the importance of both different and correlated initial phases.

**Fig 5:**
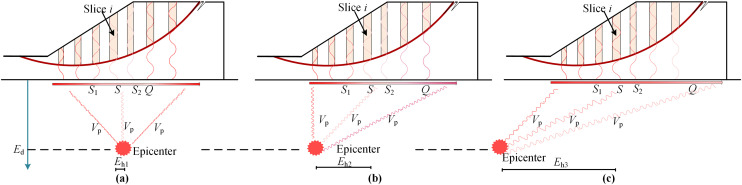
Diagram of influence of *E*_h_ on initial phase.

Fig 5 illustrates the influence of *E*_h_ on the initial phase for the given combinations of *E*_d_ and *V*_p_. As depicted in Fig 5, variations in the *E*_h_ for a specific location of the slope base accordingly result in the disparity in its initial phase. Consider slice *i* for example, its projection point in the slope base is *S.* The respective *E*_h_ of point *S* is *E*_h1_, *E*_h2_, and *E*_h3_ in Fig 5. Given the same *V*_p_, the initial phase of *S* varies accordingly. In addition, the initial phases at adjacent points *S*_1_ and *S*_2_ correlate to that at point *S*. This correlation weakens as the distance between point *S* and the focus point, see point *Q*, for example increases. It is intuitively derived from Fig 5 that the correlation in initial phase at point *S* and *Q* is sorted in descending order as the distance between *S* and epicenter increases.

**Fig 6:**
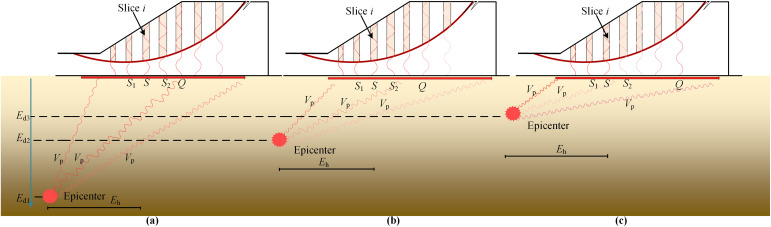
Diagram of influence of *E*_d_ on initial pahse.

**Fig 7:**
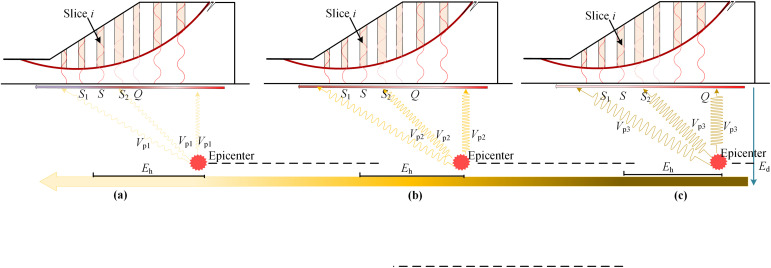
Diagram of influence of *V*_p_ on initial phase.

Apart from *E*_h_, *E*_d_ which ranges from several hundred meters to tens of kilometers exhibits significant influence on the initial phase [32]. Fig 6 demonstrates the variation trend of initial phase at point *S* and the correlation trend of initial phases between point *S* and its adjacent points *S*_1_ and *S*_2_, and *Q* as well under the influence of *E*_d_. Refer to Fig 6, for the same *E*_h_ of point *S*, the *E*_d_ is *E*_d1_, *E*_d2_, *E*_d3_, respectively. Given the same *V*_p_, the initial phase of *S* varies accordingly. It is noticed from Fig 7 that for the same *E*_h_ and *E*_d_ of point *S*, the respective *V*_p_ is *V*_p1_, *V*_p2_, *V*_p3_ in Fig 7. Given the same *E*_h_ and *E*_d_, the initial phase of *S* varies accordingly. It can be concluded from Figs 5–7 that the initial phase of point *S* is different but highly correlated with those of adjacent points *S*_1_ and *S*_2_, and the correlations diminish as the relative distance between two points increases. The variation and correlation trends of initial phases coincide with those concepts of random field theory, where the soil properties at two different locations are different but correlated and the correlation decreases as the relative distance between two locations increases [33]. To properly simulate both different and correlated initial phases, the random field theory will be briefly reviewed in the next section.

### 3.2 Simulation of correlated initial phases using random field theory

The random field theory was originated by Vanmarcke [33] to simulate the spatial variability of soil properties in geotechnical engineering. Since the initiation of random field theory, it has been widely used to investigate the influence of spatial variability of soil properties on the performance of geotechnical structures, e.g., slope, foundation, and embankments [33–45]. The fundamentals of the random field theory are the autocorrelation function and the scale of fluctuation. Since the precise determination of the autocorrelation function is nontrivial, the previous studies [46–52] can be referred to for preliminary studies. As for the determination of the scale of fluctuation for the initial phase, in situ or laboratory model tests can be referred to, where the accelerations of at different locations on the slope base are to be measured.The determination of the autocorrelation function and scale of fluctuation is not given in this study and the influence of scale of fluctuation of the initial phase on seismic slope stability is focused on. Consider the stationary random field of initial phase for example, the exponential autocorrelation function is assumed for simplicity and the scale of fluctuation, denoted by *λ*, is selected to reflect the integrated influence of *E*_d,_ *E*_h_,and *V*_p_ on the correlation of initial phases. The initial phase, denoted by *P*, is modeled by one dimensional random field spatially varying along the horizontal direction. The value of *P* at the same width is assumed to be fully correlated. The spatial variability with width is simulated by a homogeneous uniform random field with an exponentially decaying correlation structure.

**Fig 8:**
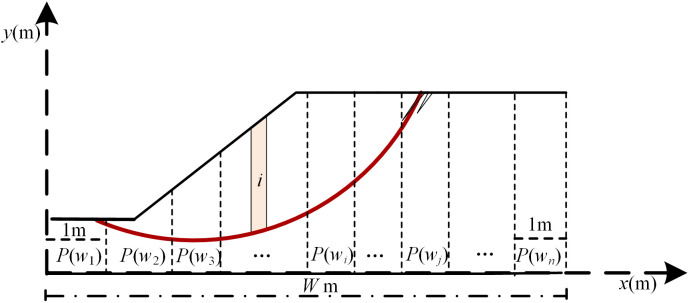
Discretization of random field variable *P.*


To generate the correlated initial phases using the random field theory, the mid-point method [39,53,54] is adopted to discretize the slope base domain. As shown in Fig 8, the *W*-m-wide slope base is divided into *n* 1-m-wide sub-bases. The initial phase within each sub-base is represented by an entry in a **P** vector with a length of *W*. *P* is assumed to be a uniformly distributed random variable within lower limit *I*_l_ and upper limit *I*_u_. Let *P*(*w*_*i*_) be the initial phase at width *w*_*i*_ and $\Phi^{-1}\left(.\right)$ be the inverse cumulative distribution function. The correlation *η*_*ij*_ between $\Phi^{-1}\left(\frac{P\left(w_{i}\right)}{I_{u}-I_{l}}\right)$ and $\Phi^{-1}\left(\frac{P\left(w_{j}\right)}{I_{u}-I_{l}}\right)$ at respective width *w*_*i*_ and *w*_*j*_ can be given by:

$$\eta_{ij}=\exp(-2\left|w_{i}-w_{j}\right|/\lambda )$$ (11)

where *λ* is the scale of fluctuation. when $\left|w_{i}-w_{j}\right|/\lambda \geq 1$ , $\Phi^{-1}\left(\frac{P\left(w_{i}\right)}{I_{u}-I_{l}}\right)$ and $\Phi^{-1}\left(\frac{P\left(w_{j}\right)}{I_{u}-I_{l}}\right)$ are effectively uncorrected [34]. In contrast, when $\left|w_{i}-w_{j}\right|/\lambda <<1$,$\Phi^{-1}\left(\frac{P\left(w_{i}\right)}{I_{u}-I_{l}}\right)$ and $\Phi^{-1}\left(\frac{P\left(w_{j}\right)}{I_{u}-I_{l}}\right)$ are highly correlated. In this example, the value of *λ* varies from 4 m to infinity for consideration of different spatial correlations.

Consider, for example, covariance matrix decomposition method is adopted to generate the horizontal uniform random field of *P*, denoted by R(P).


$$R(P)=I_{1}+(I_{u}-I_{1})\Phi (L\theta )$$ (12)

where $\theta ={\left[\theta_{1},\;\theta_{2},\;...,\theta_{n}\right]}^{T}$ **=** a standard Gaussian vector with *n* independent components; L = a *n*-by-*n* lower-triangular matrix determined from Cholesky decomposition of the correlation matrix η **=** $\;LL^{T}$.

## 4. The proposed methodology

**Fig 9:**
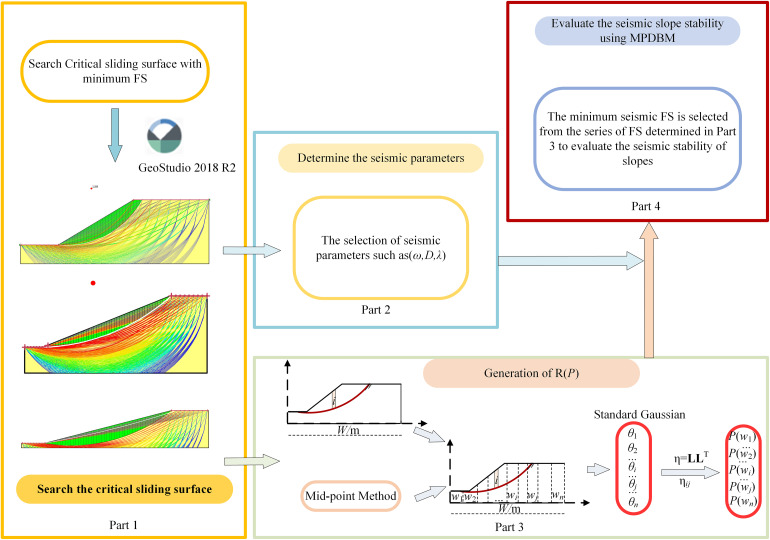
Flowchart of MPDBM.

The proposed methodology, which combines PDBM with correlated initial phase **R**(**P**) to assess the seismic stability of soil slopes, is called MPDBM. Fig 9 depicts the flowchart of the proposed methodology. It is seen from Fig 9 that MPDMB consists of four parts. That is, Part 1:Search the critical sliding surface; Part 2: Determine the seismic parameters; Part 3: Generation of **R**(**P**); Part 4: Evaluate the seismic slope stability using MPDBM.

In Part 1, the slope model is established in Geostudio and the critical sliding surface with minimum FS is searched, based on which the seismic slope stability is evaluated. Part 2 involves the selection of seismic parameters such as the angular frequency of seismic wave *ω*, the shear wave velocity in the slope soils *V*s, the damping ratio *D* and scale of fluctuation in the random field of **R**(**P**). Part 3 is the generation of **R**(**P**), based on which a series of seismic FS can be calculated using the MPDBM. In Part 4, the minimum seismic FS is selected from the series of FS determined in Part 3 to evaluate the seismic stability of slopes.

## 5. Validation of MPDBM

Before the proposed MPDBM can be extended to case studies, it should be validated against the results from the previous research [16]. The maximum active thrust acting on the rigid retaining wall and the corresponding distribution of horizontal seismic acceleration were investigated using the MPDM in [16]. Since the distribution of horizontal seismic acceleration is dependent only on *ω*, *V*s, *H*, and *D*, the slope stability problem is adopted to investigate the distribution of horizontal seismic acceleration leading to minimum FS, which are comparable with the results from [16].

**Fig 10:**
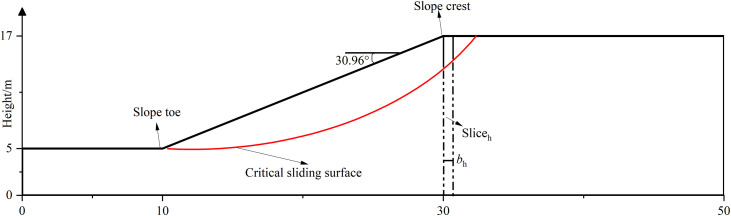
The slope model and the specific vertical slice.


As shown in Fig 10, a homogeneous slope with slope height of 12m and slope angle of 30.96° is considered. The unit weight of the slope soil is 18kN/m^3^. The respective cohesion and frictional angle are 8 kPa and 20°. As a preliminary approach to evaluate the seismic slope stability, the slope stability without considering seismic forces is encouraged. In this paper, the Bishop method is selected in SLOPE/W to calculate the FS for the specific circular sliding surface. The circular sliding surface with minimum FS is defined as the critical sliding surface. The minimum FS without seismic forces is determined to be 1.104 and the critical sliding surface is plotted in Fig 10 in red line. The proposed MPDBM is used to calculate the minimum seismic FS of the critical sliding surface and the corresponding distribution of seismic horizontal acceleration. To simplify the comparison, a specific vertical slice with a width of *b*_h_, denoted by slice_h_ in Fig 10 is focused on to study the distribution of horizontal seismic acceleration with the normalized depth. To ensure identical comparison with the result from [16], the same parameters are adopted.That is, *Nf* = 2, and *D* = 0.1, *k*_h_ = 0.2. The normalized frequency(*Nf*) is defined as:

Nf=ωH/Vs (13)

**Fig 11:**
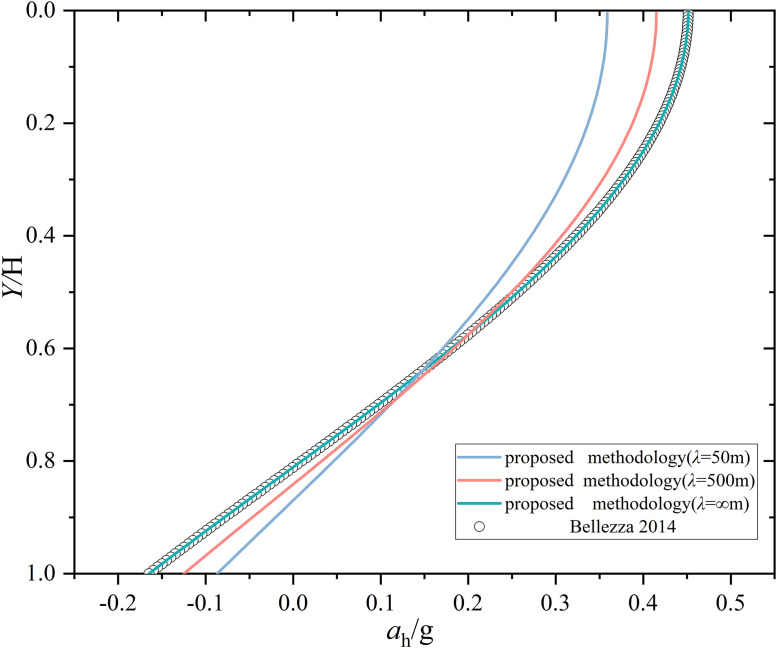
Distribution of horizontal acceleration versus normalized depth.

The 100 realizations of **R**(**P**) are generated following the descriptions in Section 3 under a set of scale of fluctuations(λ = 50, 500 and +∞m). Refer to Section 3, the slope base is 50m wide and it is discretized into 50 1-m-wide sub-bases. Each of 100 realizations of **R**(**P**) is regarded as the input for the initial phases of 50 sub-bases. The corresponding FS is calculated using Eq.(10). As a result, 100 FSs are obtained and the minimum one is found to be 0.701,0.689 and 0.683 under *λ* = 50, 500 and +∞m, respectively. The realization of **R**(**P**) leading to the minimum FS is plotted versus the normalized depth. Fig 11 summarizes the distributions of horizontal seismic accelerations (*a*_h_) under *λ* = 50, 500, and +∞m, respectively and the results form the Bellezza (2014) as well. As shown in Fig 11, the distribution of *a*_h_ obtained at *λ*=+∞m (i.e., the initial phases are fully correlated as in MPDM) agrees well with that from [16]. The consistent comparison demonstrates the effectiveness of the proposed MPDBM. That is, the proposed MPDBM degenerates into MPDM when the spatial variability is not considered (*λ=*+∞m). As *λ* decreases,i.e., the spatial variability of initial phases turns to be significant, the distribution of horizontal seismic acceleration differs from that without considering spatial variability of initial phases.

## 6. Case studies

The proposed methodology is illustrated through the homogeneous soil slope shown in Fig 10. The critical sliding surface depicted in red line in Fig 10 is based on to evaluate the seismic stability of the slope. How the variation of critical sliding surface influences the seismic stability will be discussed in Section 6.5. For seismic parameters, the *k*_h_ = 0.1 and *D* = 0.2 are specified.

### 6.1 Determine the minimum FS in MPDM using uniform sampling approach

Following the previous research outputs of MPDM [16,27], The normalized frequency is reckoned as a crucial factor. Bellezza [16] has pointed out that *Nf* = 0.5π, where the slope soil is subjected to its natural frequency, leads to maximum seismic forces and minimum FS. Hence, at *Nf* = 0.5π, the variations of FS(*t*) with initial phase *t* is demonstrated and an equivalent uniform sampling approach, which is easily implemented into MPDBM, is described and validated. Consider *ω* = 2π (i.e.,*T* = 1s), the initial phase *t* in Eq.(10) is assumed to be a set of values ranging between 0 and *T* with equal increment ∆*T*. Equation (10) is used to calculate the FS(*t*) under *Nf = *0.5π, 1.5π, and 2.0π, respectively.

**Fig 12:**
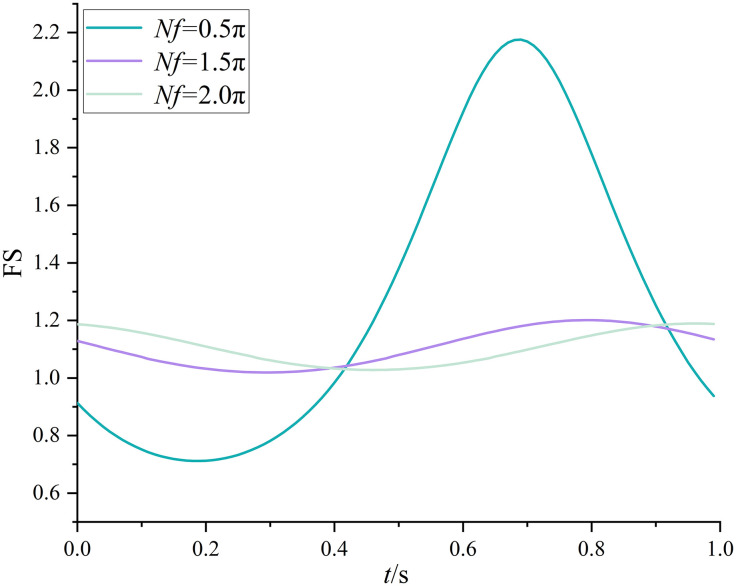
Variation of FS(*t*) with *t.*

**Fig 13:**
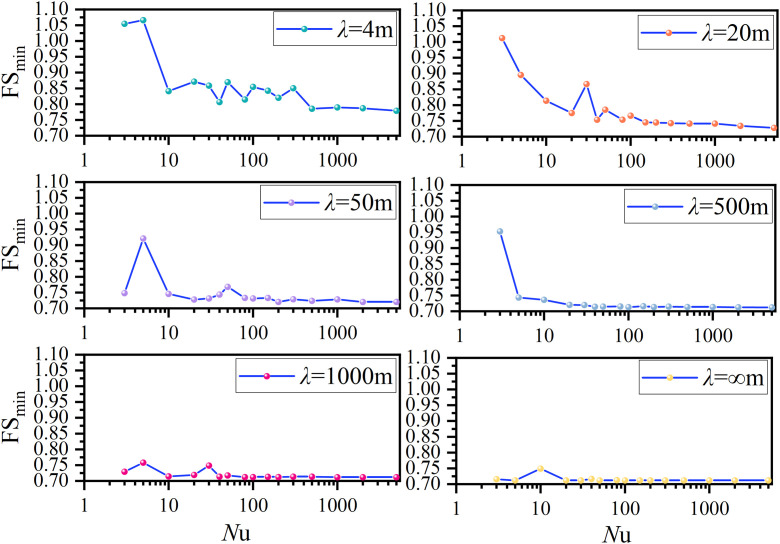
Variation of FS_min_ with *N*u for different *λ*s at *Nf* *=* 0.5π.

The respective variations of FS(*t*) with *t* under *Nf = *0.5π, 1.5π, and 2.0π are plotted in Fig 12. For the case of *Nf = *0.5π, the FS(*t*) decreases from 0.91 to a minimum of 0.71 as *t* varies from 0 to 0.19, and it increases to to a maximum of 2.18 as *t* ranges from 0.19 to.0.69, and finally it decreases to the original value of 0.91(*t* = 0) as *t* increases from 0.69 to 1.0. This variation trend indicates that the FS(*t*) is a periodical function with a period of *T* = 1s. The minimum FS value 0.71 is adopted for assessing the slope stability. Similar variation trends have been noticed for the cases of *Nf = *1.5π, and 1.5π. It is clearly noticed that the respective minimum FS values of FS(*t*) are 0.71, 1.02 and 1.03 with *t* = 0.19s, 0.29s and 0.46s under *Nf = *0.5π, 1.5π, and 2.0π. It is demonstrated that the minimum FS value and the corresponding initial phase *t* are dependent on *Nf.* The determination of minimum FS value is of importance. An mathematical method, where the derivative of active thrust to initial phase *t* is adopted, is presented in [16]. However, in this study, refer to Eq.(10), the derivative of FS to initial phase *t* is not easily dealt with as compared to that in [16]. Therefore, a set of tentative initial phase *t* must be tried and compared to find the minimum FS value as does in Fig 12. To facilitate the implementation of MPDBM, an alternative approach, named uniform sampling approach, is proposed in this study to find the minimum FS value and the corresponding initial phase *t*. The initial phase *t* is assumed to be a uniformly distributed random variable within the range between 0 and *T*. A finite number of random samples are generated within the range between 0 and *T*, consider *N*_u_ random samples for example, each of *N*_u_ samples is substituted into Eq.(10) and a FS can be obtained. Finally, a total of *N*_u_ FS values are available. The minimum one among the *N*_u_ FS values is the minimum FS value. Fig 13 demonstrates the variation of FS_min_ with different *N*_u_ values at different *λ* under *Nf = *0.5π.

Consider *λ* = 4m as an example, the respective FS_min_ is 1.05, 1.07, 0.84, 0.87, 0.86, 0.80, 0.86, 0.81, 0.85, 0.84, 0.82, 0.85, 0.79, 0.79, 0.79 and 0.79 for *N*_u_ = 3, 5, 10, 20, 30, 40, 50, 80, 100, 150, 200, 300, 500, 1000, 2000 and 5000. It is noticed that once *N*_u_ is greater than 500, the FS_min_ remains unchanged at 0.79. As *λ* increases, e.g., *λ* = 20m*,* the respective FS_min_ is 1.01, 0.89, 0.81, 0.77, 0.87, 0.75, 0.79, 0.75, 0.76, 0.74, 0.74, 0.74, 0.74, 0.74, 0.74 and 0.74 for *N*_u_ = 3, 5, 10, 20, 30, 40, 50, 80, 100, 150, 200, 300, 500, 1000, 2000 and 5000. When *N*_u_ is greater than 200, the FS_min_ remains unchanged at 0.74. For the rest of *λ* cases, similar variation trends have been noticed. However, it is worthy to note that a larger *λ* relates to a smaller minimum *N*_u_ beyond which the FS_min_ obtained by uniform sampling approach is not varied. It implies that as the spatial variability of initial phases grows significant, more samples are required in uniform sampling approach, and vice versa. When it comes to an extreme case, i.e., *λ=*∞m, the minimum *N*_u_ is 35 and the final unfluctuated FS_min_ is 0.71, which coincides with that found in the traditional method shown in Fig 12. This identical comparison justifies the effectiveness of the uniform sampling approach. As a result, the uniform sampling approach is adopted in MPDBM.

### 6.2 Effect of *λ* on seismic slope stability

The proposed methodology is used to calculate the minimum FS value for a variety of *λ* values in order to investigate the effect of *λ* on seismic slope stability. A set of *λ* values, e.g., 4, 20, 50, 500, 1000,and +∞ are assumed. Starting with an initial value of 0.1π, *Nf* is increased by equal step of π/200 until it reaches 2π, leading to a total of 400 individual *Nf* values. For each combination of *λ* and *Nf,* following the description in Section 3.2, the-50-m-wide slope base is divided into *n = *50 1-m-wide sub-bases, Equation(11) is used to establish the correlation matrix **L**, and finally *Nu* samples (i.e., *Nu* realizations of **R**(**P**)) are generated in accordance with Eq.(12). It is noted that *I*_1_ = 0 and *I*_u_* = T* in this study. Thereafter, the proposed uniform sampling approach is used to calculate the minimum FS value. The full combinations of six *λ* values and 400 *Nf* values yield 2,400 minimum FS values.

**Fig 14:**
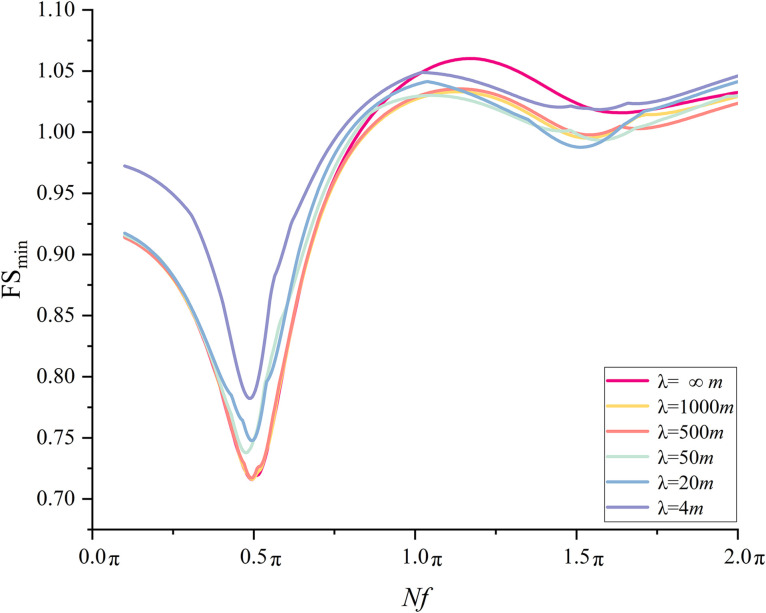
Variations of FS_min_ with *Nf* at different *λ*s (small scale slope).

**Fig 15:**
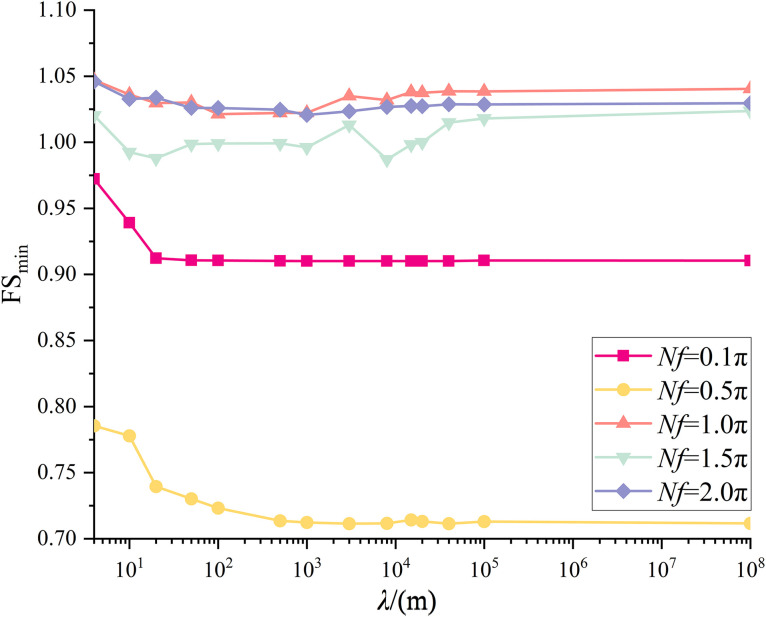
Variations of FS_min_ with *λ* at different *Nf*s (small scale slope).

The results are summarized in Fig 14. For *λ* = ∞ m, the respective FS_min_ is 0.91, 0.71, 1.04, 1.02, and 1.03 under *Nf* = 0.1π, 0.5π, 1.0π, 1.5π, and 2.0π. Two local minima are observed. One is found at *Nf* = 0.5π and the other is at 1.5π, which is consistent with the observations from MPDM in [16,27]. The minimum FS_min_ is 0.71 at *Nf* = 0.5π. Quite similar variation trends of FS_min_ with *Nf* are noticed for the cases of *λ* = 4, 20, 50, 500, and 1000 m. However, the respective minimum FS_min_ are 0.79, 0.75, 0.74, 0.72 and 0.72 at *Nf* = 103π/200, 99π/200, 19π/40 and 99π/200 for *λ* = 4, 20, 50, 500 and 1000m. It is worth noting that the respective minimum FS_min_ is in the vicinity of 0.5π. To explore the relationship between *λ* and the minimum FS_min_ in detail, Fig 15 depicts the FS_min_ for different *λ* values at *Nf* = π/10, π/2, π, 3π/2, 2π. For example, at *Nf = *π/2, the respective FS_min_ is 0.79, 0.74, 0.72, 0.71, 0.71for *λ* = 4, 20, 50, 500, 1000 and ∞ m. It is observed that as *λ* increases from 4m to 500m, the FS_min_ decreases dramatically from 0.79 to 0.71, and it varies slightly around 0.71 as *λ* is greater than 500m. The difference between 0.79(*λ* = 4m) and 0.71(*λ*=*+*∞m) can be up to 11%, which significantly underestimates the seismic slope stability and tends to result in a conservative slope design if spatial variability of initial phase is not considered.

It is worth noting that the variation trend of FS_min_ with respect to *λ* is likely to differs from that at *Nf* = 0.5π. For example, as illustrated in Fig 15, at *Nf = *3π/2, the respective FS_min_ is 1.02, 0.99, 0.99, 0.99, 1.00 and 1.02 for *λ* = 4, 20, 50, 500, 1000 and ∞ m. It is seen that the FS_min_ drops firstly and then it increases to a value close to the initial value. The minimum FS_min_ is observed at *λ* = 20m, which is clearly different from that observed at *Nf* = 0.5π(the minimum FS_min_ is at *λ* = +∞m). The results from the MPDBM without considering spatial variability of initial phases can be either conservative or unconservative depending on the *Nf* value.

### 6.3 Effect of *D* on seismic slope stability

**Fig 16:**
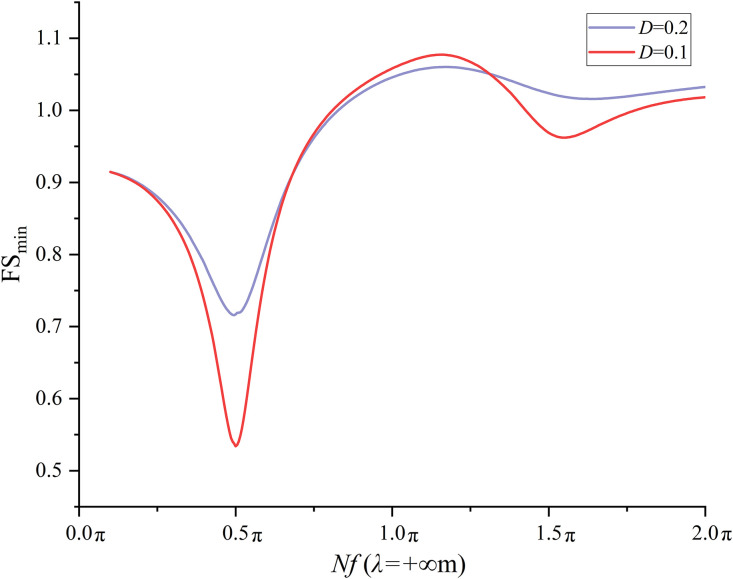
Effect of *D* on FS_min_ at *λ*=∞m.

**Fig 17:**
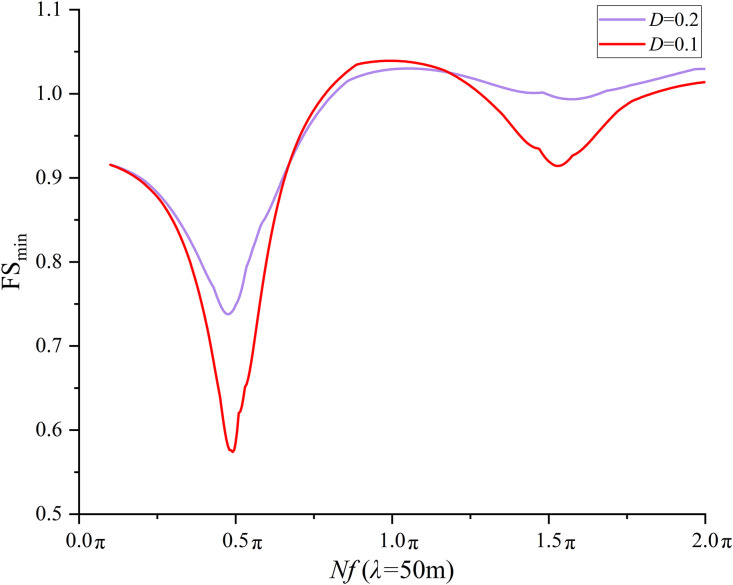
Effect of *D* on FS_min_ at *λ* = 50m.

**Fig 18:**
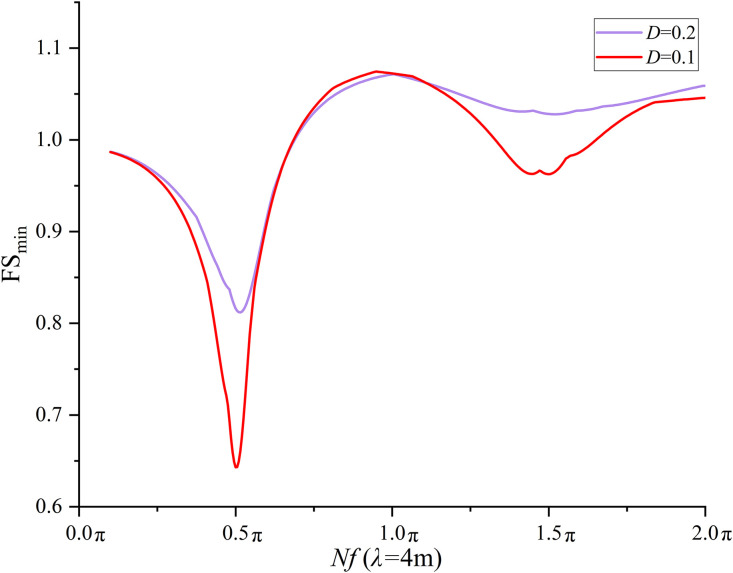
Effect of *D* on FS_min_ at *λ* = 4m.

*D* is assumed to be constant at 0.2 in the previous sections 6.2 and 6.3. To investigate the effect of *D* on the seismic slope stability, *D* = 0.1 is specified and the FS_min_ at each combination of three *λ*(4, 50, and +∞m) and 400 *Nf* values is calculated. The results from *D* = 0.2 are compared with those from *D* = 0.1. Fig 16 compares the variation trend of the FS_min_ with respect to *Nf* for *D* = 0.1 and 0.2 at *λ* = +∞ m. It is observed from Fig 16 that the variation trend of FS_min_ at *D* = 0.1 agrees fairly well with that at *D* = 0.2. It is interesting to note that the FS_min_ calculated at *D* = 0.1 is lower than that at *D* = 0.2 as *Nf* ranges between 0.1 and 0.685π. The most significant difference is found at *Nf* = 0.5π (the *Nf* leading to the first local minimum), where the difference in FS_min_ reaches 0.19 = 0.72-0.53.As *Nf* varies from 0.685π to 1.31π, the FS_min_ calculated at *D* = 0.1 is slightly greater than that at *D* = 0.2 with a maximum difference of 0.02 in FS_min_ at *Nf* = 1.145π. When *Nf* increases from 1.145π to 2.0π, the FS_min_ calculated at *D* = 0.1 is lower than that at *D* = 0.2 with a maximum difference of 0.05 = 1.02-0.97 in FS_min_ at *Nf* = 1.5π. The comparisons at *λ* = 50 m and *λ* = 4m are shown in Fig 17 and Fig 18, respectively. Very similar variation trends have been noticed at *λ* = 4 and 50 m. It is concluded from the comparisons that a smaller *D* tends to yield a lower FS_min_ especially at *Nf* values corresponding to the natural frequency of slope soil. It must be noted that a smaller *D* also leads to a slightly greater FS_min_ when *Nf* ranges between 0.5π and 1.5π in this study.

### 6.4 Effect of λ on seismic stability of slope with different scale

**Fig 19:**
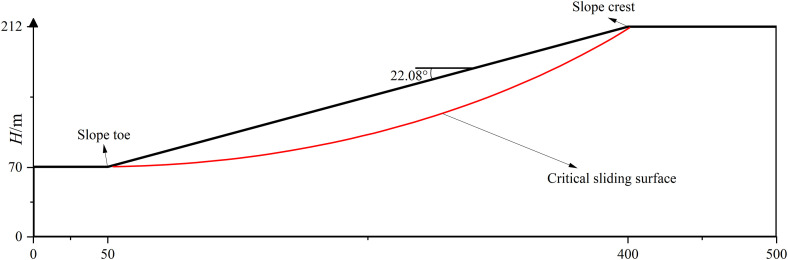
Homogeneous soil slope(medium scale).

**Fig 20:**
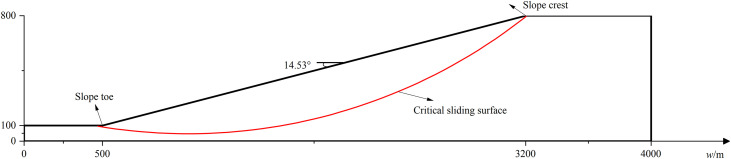
Homogeneous soil slope(large scale).

Two slopes with different scales are considered in this section. The first one is shown in Fig 19. It is a medium scale homogeneous slope with slope height** = **212m and slope angle = 22.08°.The cohesion *c*, internal friction angle *φ*, soil unit weight and damping ratio of soil are 18 kPa, 20°, 18*k*N/m^3^ and 0.2,respectively. The other one, a large scale slope, has a slope height = 800m and slope angle = 14.53°,which is shown in Fig 20. The cohesion *c*, internal friction angle *φ*, soil unit weight and damping ratio of soil are 20 kPa, 16°, 19*k*N/m^3^ and 0.2,respectively. Similarly, the Bishop method is used to calculate the FS for a given slip circular surface.The respective minimum FS without seismic forces is determined to be 1.10 and 1.21 and the corresponding critical sliding surfaces are plotted in red line in Figs 19 and 20 for the medium and large scale slope.

**Fig 21:**
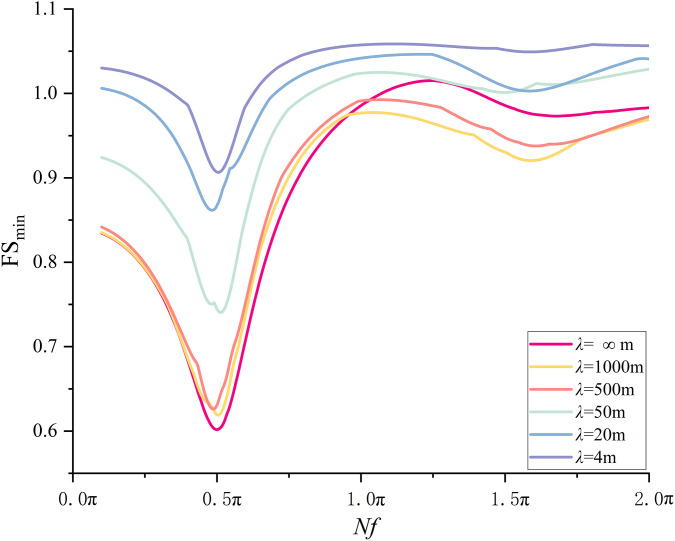
Variations of FS_min_ with *Nf* at different *λ*s for medium scale slope.

**Fig 22:**
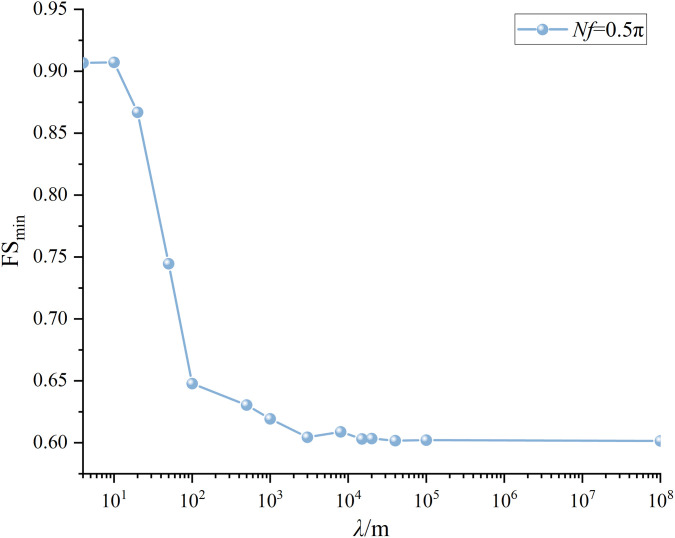
Variations of FS_min_ with *λ* at *Nf* = 0.5π for medium scale slope.

**Fig 23:**
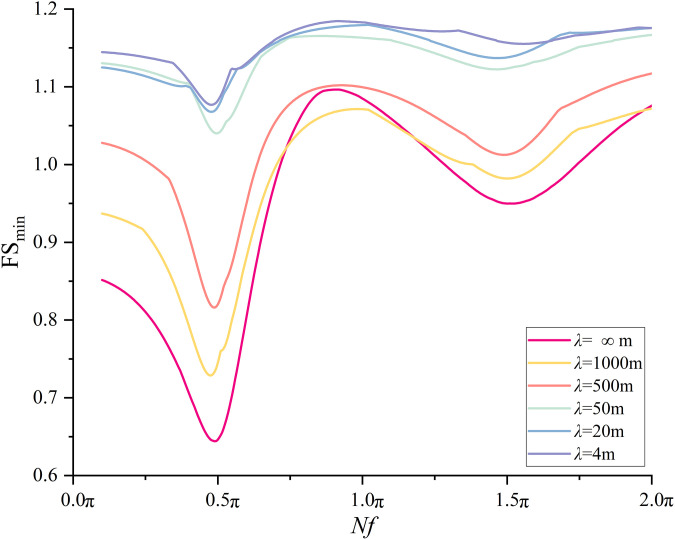
Variations of FS_min_ with *Nf* at different *λ*s for large scale slope.

**Fig 24:**
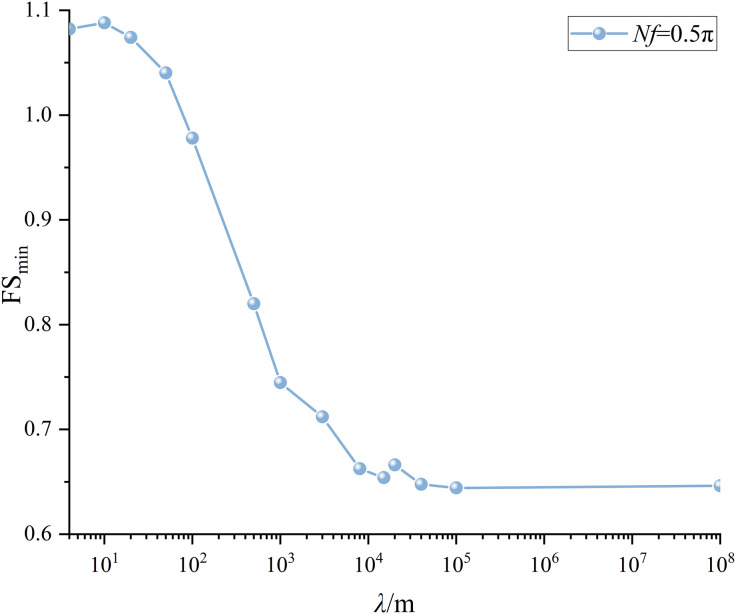
Variations of FS_min_ with *λ* at *Nf* = 0.5π for large scale slope.

Similar to the treatment in Section 6.2, 2400 combinations of *λ* and *Nf* are considered. The variation trends of FS_min_ with *Nf* and *λ* are shown in Figs 21–24 for two slopes with different scales. It can be noticed that the variation trends found in Figs 21 and 23 agree with those demonstrated in Fig 14. Fig 22 plots the variation of FS_min_ with *λ* under *Nf* = 0.5πfor the medium scale slope. It is seen that **t**he FS_min_ drops from 0.91 to 0.60 as *λ* values increase from 4 to +∞m for medium scale slope. The difference between 0.91(*λ* = 4m) and 0.60(*λ*=*+*∞m) reaches 50%, which is significantly higher than 14% as compared to small scale slope. This comparison indicates a more pronounced effect of *λ* on medium scale slope.

Fig 24 plots the variation of FS_min_ with *λ* under *Nf* = 0.5π for the large scale slope. In the case of large scale slope, the FS_min_ drops from 1.08 to 0.65 as *λ* values increase from 4 to +∞m. The discrepancy between 1.08(*λ* = 4m) and 0.65(*λ*=*+*∞m) arrives at 67%, which is significantly higher than 11% (small scale slope) and slightly higher than 50%(medium scale slope). The effect of of *λ* on seismic slope stability grows significant as the slope scale increases. As a result, the proposed MPDBM is recommended for seismic stability analysis of slopes with medium and large scale slopes.

### 6.5 The influence of critical sliding surface

**Fig 25:**
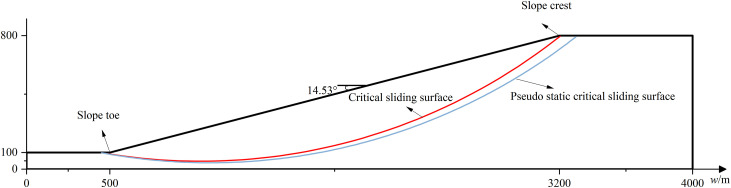
Locations of different critical sliding surfaces of large scale slope.

In the previous sections, the critical sliding surface without seismic forces is focused. To see how the varied critical sliding surfaces influence the seismic stability, the critical sliding surface with pseudo static forces (*a*_h_ = 0.1g), defined as pseudo static critical sliding surface is incorporated into the analysis. The locations of the two mentioned critical sliding surfaces are compared in Fig 25. The FS_min_s for the pseudo static critical sliding surface at each combination of two *λ*(4 and +∞m) and 400 *Nf* values are calculated.

**Fig 26:**
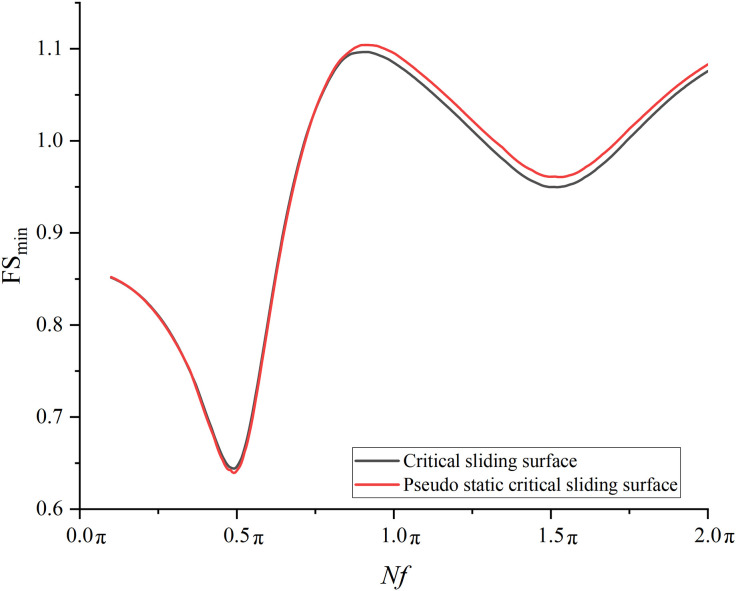
Variations of FS_min_ with *Nf* for large scale slope under different slip surfaces at *λ* = +∞.

**Fig 27:**
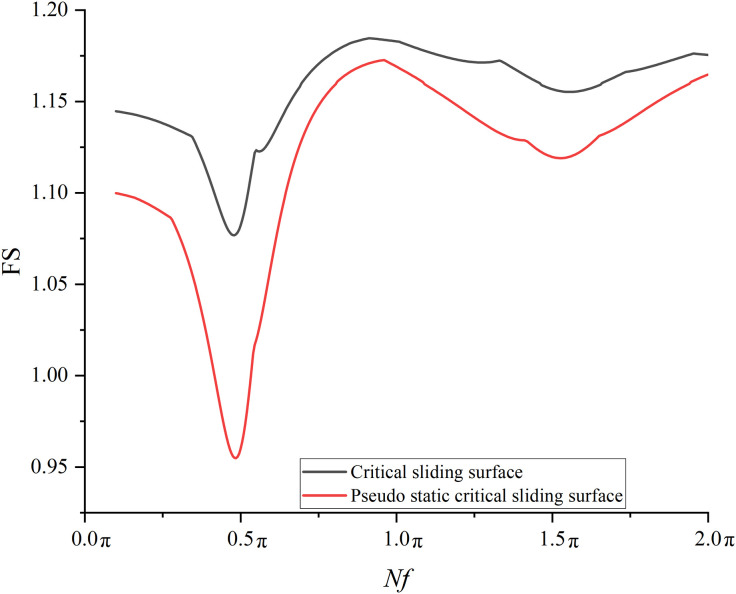
Variations of FS_min_ with *Nf* for large scale slope under different slip surfaces at *λ* = 4m.

The results from critical sliding surface without seismic forces are compared with those from pseudo static critical sliding surface. Fig 26 compares the variation trends of the FS_min_ with respect to *Nf* at *λ* = +∞ m between critical sliding surface without seismic forces and pseudo static critical sliding surface. It is observed from Fig 26 that the variation trend of FS_min_ for pseudo static critical surface closely aligns with that for critical surface without seismic force. However, it is noted from Fig 27 that significant discrepancy in FS_min_ has been observed for each of *Nf*s at *λ* = 4 m. Specifically,when *Nf* = 0.5π, the FS_min_ from critical sliding surface without seismic forces is 1.088, whereas it decreases to 0.968 if the pseudo static critical sliding surface is used. Therefore, it must be noted that the critical sliding surface should be carefully selected. Based on the current comparisons, it is found that the choice of critical sliding surface tends to significantly influence the seismic stability at *λ* = 4 m.

## 7. Discussion

The proposed MPDBM emphasizes two issues in the use of pseudo dynamic method for assessing seismic slope stability. The first one is how to find the initial phase leading to the minimum FS for a given sliding surface. The other one is how to consider both the difference and the correlation in the initial phases. To address the first issue, a uniform sampling approach is originated and validated as an alternative to the traditional method, where a finite number of individual initial phases are tried. The advantage of the uniform sampling approach beyond the traditional method lies in its time efficiency, especially for low frequency seismic waves. It is interesting to note that the first issue has scarcely been dealt with. One exception is the research from [16], where a mathematical approach is adopted to directly determine the initial phase. Compared to the mathematical approach, the uniform sampling approach exhibits broader applicability because it does not require mandatory derivatives of geotechnical performance indicators (e.g., FS, displacement) with respect to the initial phase.

The second issue stems from the uncertainties and randomness in seismic motions. The seismic uncertainty is fairly complicated and only epicentral horizontal distance, epicentral depth, and the propagation velocity of the seismic wave in the slope base is briefly discussed to illustrate the existence of second issue. The initial phase behaves somewhat like the spatial variability of soil properties owing to the fact that both the difference and the correlation in the initial phase must be properly dealt with. Therefore, uniform random field of initial phase is adopted herein to simulate the spatial variability in the horizontal direction along the slope base. It is worth noting that the initial phase is assumed to be uniformly distributed for preliminary analysis. Besides, the uniform sampling approach and the uniform random field of initial phase coincide in the uniform essence of the initial phase.

For simplicity, the circular sliding surface and the simplified Bishop method are adopted to evaluate the seismic slope stability for simplicity and for the homogeneous soil slopes. However, the uniform sampling approach and the uniform random field can be easily combined with existing methods for assessing slope stability including but not limited to limit equilibrium methods, limit analysis, and strength reduction method. As a result, the proposed methodology is versatile although only homogeneous soil slopes are discussed in this study.

The proposed MPDBM is implemented within a commercial software package, SLOPE/W, and effects of the scale of fluctuation, damping ratio, and the slope scale on the seismic FS are investigated, providing a basis for further analysis of key findings.

## 8. Conclusions

The proposed MPDBM is developed to address the limitations of traditional pseudo dynamic methods in accounting for initial phase variability, and its application and key findings are summarized as follows:

(1) The proposed MPDBM is able to delineate the difference in seismic FS attributed to the spatial variability of initial phase and serve as an effective and alternative tool for conducting seismic slope stability using pseudo dynamic method.
(2) The traditional method ignoring the spatial variability of initial phase underestimates the FS for the cases where the slope soil is subjected to its natural frequency as compared to the proposed MPDBM with scale of fluctuation = 4m(i.e., the spatial variability of initial phase is significant). The underestimation turns to be profound for large scale slopes.
(3) For the cases, where the frequency of the seismic wave deviates from the natural frequency of slope soil, the consideration of the spatial variability of initial phase may yield smaller FS than the traditional method ignoring the spatial variability of initial phase.

## Supporting information

S1 File Supporting information mainly includes the data of figure. (XLSX)
